# A prosocial function of head-gaze aversion and head-cocking in common marmosets

**DOI:** 10.1007/s10329-022-00997-z

**Published:** 2022-07-15

**Authors:** Silvia Spadacenta, Peter W. Dicke, Peter Thier

**Affiliations:** Cognitive Neurology Laboratory, Hertie Institute for Clinical Brain Research, University of Tübingen, Tübingen, Germany

**Keywords:** Common marmoset, Eye contact, Gaze aversion, Social interaction, Peekaboo, Head-cocking

## Abstract

Gaze aversion is a behavior adopted by several mammalian and non-mammalian species in response to eye contact, and is usually interpreted as a reaction to a perceived threat. Unlike many other primate species, common marmosets (*Callithrix jacchus*) are thought to have a high tolerance for direct gaze, barely exhibiting gaze avoidance towards conspecifics and humans. Here we show that this does not hold for marmosets interacting with a familiar experimenter who suddenly establishes eye contact in a playful interaction (peekaboo). Video footage synchronously recorded from the perspective of the marmoset and the experimenter showed that the monkeys consistently alternated between eye contact and head-gaze aversion, and that these responses were often preceded by head-cocking. We hypothesize that this behavioral strategy helps marmosets to temporarily disengage from emotionally overwhelming social stimulation due to sight of another individual’s face, in order to prepare for a new round of affiliative face-to-face interactions.

**Supplementary Information:**

The online version contains supplementary material available at 10.1007/s10329-022-00997-z.

## Introduction

Establishing eye contact is a highly communicative act that shapes the social interactions of both humans and non-human primates (Kleinke 1986). Most primates perceive direct gaze as a display of threat that precedes an attack (Hinde and Rowell 1962; Redican 1975; Coss 1978; Mendelson 1982; Maestripieri 1997; Coss et al. 2002; Kaplan and Rogers, 2002), although eye contact of brief duration can also occur in prosocial contexts, such as courtship, cooperative actions and play (De Waal and Yoshihara 1983; Yamagiwa 1992; Sato and Nakamura 2001; Kano et al. 2015; Miss and Burkart 2018). Eye contact, when sought and sustained, is more characteristic of mother-infant interactions across a wide variety of primate species, even the ones which, in adulthood, utilize direct gaze mostly in agonistic contexts (Mendelson et al. 1982; Ehardt and Blount 1984; Tronick 1989; Bard et al. 2005; Ferrari et al. 2009; Wang et al. 2020).

Irrespective of the different behavioral meanings that eye contact assumes according to species and contexts, a typical response of humans, non-human primates and many other mammalian species to eye contact is to close or avert the eyes or turn the head away after a varying period of direct gaze (Calhoun 1962; Chance 1962). This attempt to evade a direct gaze is usually called “gaze aversion” (Coss 1978, 1979). It has been suggested that, by means of this behavior (and also others aimed at covering the eyes), primates try to stop the perception of an arousing stimulus (e.g., the direct gaze of a dominant animal) in order to continue engaging in their ongoing activities, which would be negatively impacted by excessive arousal (Chance 1962). A possible complementary function of disengaging from eye contact is that this may also signal appeasement, to prevent an attack. In humans, gaze aversion is part of the normal behavioral repertoire of both adults and infants (Argyle and Dean 1965; Cook 1977) and, in line with the variable significance of mutual gaze shaped by context and those interacting in it, it may also have different meanings. The role of gaze aversion as a regulator of perceptual input is particularly compelling in human infants (Robson 1967), given their limitations to interact with their environment and to select or refuse visual stimulation. Cohn and Tronick (1983; 1987) showed that infants’ gaze aversion is part of structured cycles of engagement (eye contact, smiling, etc.) and disengagement (gaze aversion, crying, etc.) when interacting with their caregivers. Human infants exhibit gaze aversion as a reaction to the direct gaze of an emotionally unresponsive caregiver [still face experiment (Cohn and Tronick 1983; Field et al. 1986)], but also in playful interactions, for instance when playing peekaboo (Field 1981; Stifter and Moyer 1991; Reddy 2000). The notion that gaze aversion normalizes an infant’s level of arousal is suggested by the fact that looking away quickly normalizes heart rate levels when they are elevated (Field 1981). By the same token, gaze aversion may also serve as a regulator of arousal due to positive affects (Stifter and Moyer 1991). Taking a break from eye contact, which is a source of emotional stimulation (Nichols and Champness 1971; Plutchik 1980; Helminen et al. 2011, 2018), seems to allow an infant to avoid overly distressful over-excitation and to recover for a new round of soothing emotional experiences elicited by the caregiver’s facial expression and eyes.

Common marmosets, a New World species of monkey, are known to show a particular interest in other individuals’ faces (Mitchell and Leopold 2015; Nummela et al. 2019) and to readily engage in mutual gazing in prosocial contexts, such as when cooperating in joint actions (Miss and Burkart 2018). Yet the use of gaze aversion in the regulation of social interactions, as exhibited by human infants, has never been shown in common marmosets or in other New World monkeys. Indeed, common marmosets are commonly believed to make minimal use of gaze aversion, arguably because theyprovide little indication that they may experience gazing as threatening when in contact with familiar individuals. Building on the serendipitous observation of marmosets engaging in a game of peekaboo with a human agent, we present evidence that this species deploys consistent gaze aversion behavior of a kind that we believe has a prosocial function, namely a means of controlling overwhelming emotions which, if not restrained, would jeopardize the continuance of the social interaction.

## Methods

### Subjects

We tested 16 common marmosets (*Callithrix jacchus*) divided into the following groups: group 1—three females and three males, mean age 3.8 ± 2.7 years; group 2—six females and four males, mean age 5.5 ± 1.8 year), home caged at the Center for Integrative Neuroscience of the University of Tübingen. 

At the time of the study, five (Flo, Fer, Ugh, Mir, Han) of the six members of group 1 were involved in behavioral training in an experimental setup outside the animal facility. This training involved initial preparatory steps such as the animals getting used to a chair and becoming familiar with the experimental setup before being exposed to specific behavioral requests. For these animals, access to water was always ad libitum, while food intake was controlled as required by the training. The other member (Fin) of group 1, a female, had been used in the recording of behavioral data over a period of around 1 year that required head fixation. However, at the time of the study reported here on natural behavior at the marmoset facility, she was no longer a participant in these experiments, hence, in her case, water and food were ad libitum. The animals belonging to group 2 had never been trained by the experimenter, but were familiar with her due to her regular visits to the animal husbandry. All the subjects were tested within earshot of the other marmosets in the facility, but could not see them. All the subjects were born in captivity, and were kept under the following conditions: approximately 26 °C, 40–60% relative humidity, and a 12:12 h light–dark cycle.

### Experimental setting and procedure

We recorded the common marmosets’ behavioral reactions to the familiar experimenter during a game of peekaboo, a form of play in which one player hides his/her face and then suddenly reveals it again. The animals were tested in small transparent boxes (24 × 26 × 26 cm) that were permanently attached to the front of each cage, and were allowed free movement between the compartments. Only the frontal and right side of the box were entirely transparent, allowing full visibility. Importantly, for group 1, the right side of the box allowed a view of the facility’s kitchen window, through which the experimenter and the animal caretakers could be seen arriving each day to access the marmoset facility. When the animals expected to be moved to the setup for behavioral training, or heard somebody enter the kitchen, they usually went into the transparent box and directed their attention towards this window. This lead to our serendipitous observation of the head-gaze avoidance behavior, which is explored in detail in this report, when we were approaching the window before opening the door to take one of the animals out for training.

Testing was performed under two different conditions in a pseudo-randomized order: barrier or no barrier (see Supplementary file 2 and 3). In the barrier condition the human hid behind the facility’s door, and revealed her face through the window at a random pace (box—window distance, 220 cm for Fin; 284 cm for Han; and 350 cm for Flo, Fer, Mir and Ugh). In some of the sessions, we incorporated a smaller set of trials into the sequence, in which the demonstrator presented a side or back view of themselves, thus avoiding a direct gaze. In these sessions, non-direct gaze and direct gaze trials were alternated in a pseudo-randomized fashion. In the no barrier condition, the experimenter stood closer to the animals, avoided eye contact by looking down at the floor, and established eye contact by moving the head upwards (box—experimenter distance, 100 cm). 

The interaction in both conditions started when the individual marmosets were calmly and spontaneously sitting in the transparent box waiting for the experimenter to engage in eye contact. The procedure was repeated until the animal spontaneously moved from the box back into the cage. A session was defined as a block of consecutive trials. Each time the animal moved from the box back into the cage, a session was considered completed. A new session commenced when the animal returned to the box from the cage. The number of trials per session varied according to how long the animal spontaneously interacted with the experimenter. The animals usually started to enter the box less frequently roughly 30–45 min after the onset of the recordings; therefore, the recordings never lasted for more than 1 h/day. 

Group 1 (*n* = 6) was tested by one familiar experimenter in both conditions (for Fin, Flo and Fer a total of 200 repetitions per condition were recorded; for Ugh, Mir and Han, 100 repetitions). The same experimenter tested group 2 only in the no barrier condition (n = 10), given that the facility´s layout did not allow for the barrier condition in the case of every cage. As the stereotypical reaction was extremely consistent across animals, we only collected data for 20–40 trials in group 2. Additionally, we repeated the measurements, in both the barrier and no barrier condition (40 repetitions per condition per monkey), with a second experimenter who was familiar with five of the animals of group 1.

The marmosets’ behavior was recorded using one camera that faced the transparent box and a second camera, which was mounted on the same stand, that faced the window (barrier condition) or the experimenter (no barrier condition), to represent the tested animal’s perspective. The video camera recordings were made simultaneously at a frame rate of 30 Hz; OBS 23.0.2 and IC Capture 2.4 software were used to synchronize and merge the two video files for simultaneous recordings in order to identify eye contact events (see Supplementary movies).

### Video analysis and variable scores

Individual video frames were extracted from the recordings and manually inspected and quantified using tools developed in MATLAB R2019a. For each trial, we identified an “eye contact event” as the first frame in which both of the experimenter’s eyes were visible and the animal was directly looking at the experimenter’s face. The experimenter and the animals’ view were visualized in parallel (see Supplementary Fig. 1). We then calculated the time between this frame and the frame capturing the start of head-gaze aversion (gaze aversion latency), which was characterized by the monkey shifting its head away from the eye contact position in any direction. If the animal blinked right before averting its head, the point at which the eyelids closed was considered the start of head-gaze aversion (see Supplementary Fig. 1 for an example of this). Trials in which head movement was due to the monkey hearing an external sound were excluded. A second experimenter, who was not involved in the recordings, verified the measurements from a subset of trials.

We also documented the following behavioral events: head-cocking after eye contact, which preceded head-gaze aversion; whole body movement (rotation of the trunk together with the head relative to the longitudinal axis, thereby exposing the back of the animal to the experimenter) during aversion; blinking; vocalization produced at eye contact, soon after the aversion, or in between trials. Although we did not record sound, the types of vocalization could be easily identified by the movement of the mouth and were recorded by the experimenter at the end of each live session.

## Results

### Common marmosets consistently respond to eye contact with head-gaze aversion

**Fig. 1a–e:**
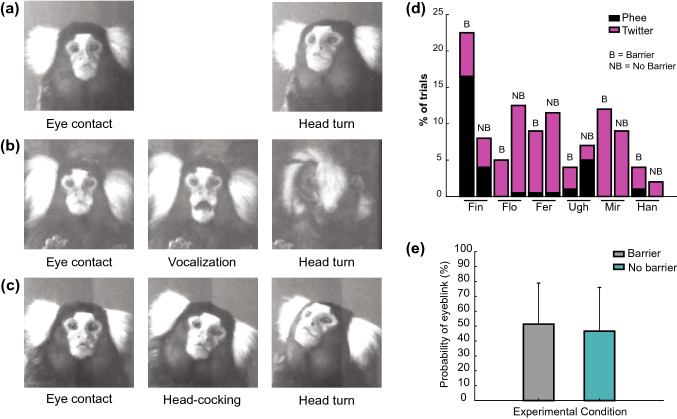
Patterns of response to eye contact, and behavioral features. **a** Simple aversion. **b** A contact call is directed at the experimenter right after eye contact is established. **c** Head-cocking (clockwise or counterclockwise) is exhibited after eye contact and before gaze aversion. **d** Percentage of trials in which a vocalization (phee or twitter) was produced. **e** Probability of blinking executed right before or at the same time as head-gaze aversion (*n* = 6)

All of the tested animals reacted to eye contact with a stereotypical pattern of head-gaze aversion regardless of the barrier condition or experimenter (Fig. 1; Supplementary Fig. 2). The initial aversive head movement was executed in different directions, typically by rotating the head 45°–90° relative to the trunk, and was coupled with a shift in gaze away from the observer. The videos did not indicate any significant counter-rotation of the eyes, suggesting a coupled shifting of the eyes and head to avert the gaze. After an interval of varying length, eye contact was resumed. In the vast majority of trials, we observed the simple aversion pattern (Fig. 1a). A second pattern, observed in a smaller percentage of trials, was characterized by the addition of a vocalization that occurred either after the animal had established eye contact with the experimenter (Fig. 1b), soon after the aversion, or in the time between periods of eye contact. In any case, the animals produced only contact calls [phees or twitters, for definitions see Chen et al. (2009); Fig. 1d). In a third pattern (Fig. 1c), head-gaze aversion could be preceded by head-cocking, i.e., rotation of the cranium along the naso-occipital axis, while maintaining a fixed direction of sight, towards the experimenter’s eyes. Moreover, independent of the specific pattern, in a large percentage of trials a blink preceded head-gaze aversion by roughly 33–66 ms (one to two video frames) or occurred at the same time (group 1, pooled percentages reported in Fig. 1e; for individual values, see Supplementary Tables 1 and 2), reminiscent of head movement-associated blinking in humans and non-human primates (Evinger et al. 1994; Tada et al. 2013) and also in a few other species [e.g., peacocks (Yorzinski 2016)]. The fact that the blink preceded the head movement in most cases speaks against the possibility that the eyelid closure might have been a protective, reflexive response to a draft that stimulated the cornea during the head turn (Evinger et al. 1994). Rather, closing the eyes might be a complimentary reaction, which supports the effect of turning the head by rapidly eliminating stimulation due to the experimenter’s direct gaze. Moreover, we have occasionally observed that many marmosets might blink not just once but several times when establishing eye contact with humans, both before and for the duration of turning the head. Whether or not blinking in this context also has a communicative function remains an open question.

Head turning was usually relatively smooth, often slow, but could be more rapid; however, regardless of how it was enacted, it was very different from the typically fast and jerky gaze shifts seen in reorientation of the position of the head in response to novel stimuli (Pandey et al. 2020). Presenting the back in conjunction with the head movement was only seen occasionally, primarily in paired animals, when the cage mate was simultaneously present in the transparent box (see Body moved in Supplementary Tables 1 and 2, for reference values).

**Fig. 2:**
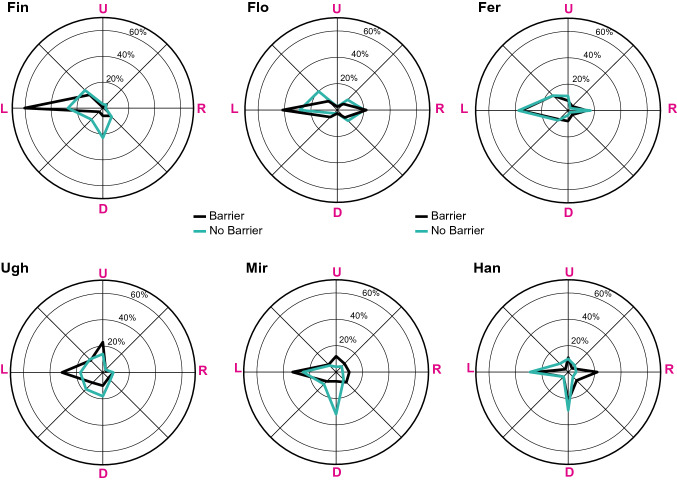
Direction of head-gaze aversion (from the animal’s perspective) in the barrier and no barrier condition. *L* Left, *R* right, *U* up, *D* down

We additionally analyzed the direction of head gaze by taking into consideration eight direction bins of 45° each in the fronto-parallel plane (Fig. 2). In general, the preferred direction for aversion was left (from the animal’s perspective), namely towards the inner part of the cage. Moreover, three animals (Fin, Ugh, Mir) showed a shift in preference towards the down direction in the no barrier condition, where the animals were exposed to the experimenter’s gaze. The results suggest that, for these animals, the final position of the turned head was influenced by the direction of the experimenter’s gaze, which, as we know from previous studies, common marmosets can follow geometrically (Burkart and Heschl 2006).

In a smaller number of trials of the barrier condition, the experimenter’s side or back was exposed to the animals, thereby avoiding direct gaze. When first exposed to these views, the monkeys did not exhibit head-gaze aversion. This is exemplified by Supplementary file 4, which compares an animal´s reaction to presentation of the side and back of the experimenter: while a direct gaze elicited multiple blinks and a subsequent turning of the head, a view of the side of the experimenter elicited no behavioral response. However, we noticed that, after a period of habituation to the demonstrator performing the movements behind the barrier, the animals could start to perform head turning, albeit, usually not as clearly, strongly and consistently as in the direct gaze condition. As these movements were typically much weaker and more variable, attempts to reliably document this behavior quantitatively was not possible. We speculate that the head turns provoked by presentation of the side or back might be a consequence of the fact that trials that did not include gazing took place in between the many more trials with direct gaze. Hence, seeing the human agent from the side or the back most probably became quickly associated with the inevitable experience of direct gaze a moment later. Thus, we surmise that there was a possible transfer of a certain degree of emotional arousal to the non-direct gaze conditions. If this interpretation is correct, one might expect that the transfer of arousal to non-direct gaze stimuli should subside quickly if no or only a few trials with direct gaze stimuli are incorporated into the trial sequence. Rigorously testing this interpretation would require experiments in which the relative proportions of direct gaze and non-direct gaze stimuli are varied, and include measurements of physiological indicators of arousal.

### Latency of aversion

**Fig. 3:**
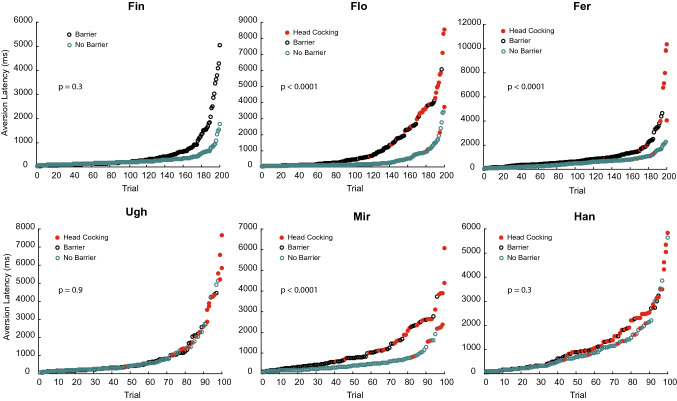
Effects of experimental conditions on aversion latency. For each monkey of group 1, the single trial aversion latency sorted by ascending duration is shown.* Red dots* Trials in which the animals either performed head-cocking or whose heads were tilted from the start of eye contact when they were looking at the experimenter. Results of the Mann–Whitney *U*-test comparing barrier and non-barrier condition latencies are reported

Head-gaze aversion latency, or eye contact duration, defined as the time between the onset of eye contact and the initiation of aversion, was in the large majority of trials less than 1 s (see Fig. 3 and Supplementary Tables 1 and 2 for median values). For group 1, we explored the effect of the presence of the barrier on aversion latency. Flo, Fer and Mir showed aversion significantly faster when the experimenter was in direct contact with them (Mann–Whitney *U*-test, Flo,* z* = 6.232, *p* < 0.0001; Fer,* z* = 4.669, *p* < 0.0001; Mir,* z* = 4.816, *p* < 0.0001), while for Fin, Ugh and Han there was no significant difference (Fin,* z* = 0.99, *p* = 0.3; Ugh,* z* = − 0.099, *p* = 0.921; Han* z* = 1.042, *p* = 0.3). When the second experimenter interacted with the animals (see Supplementary Fig. 2; Supplementary Table 3) an effect of the barrier was only shown for Flo (*z* = 2.881, *p* < 0.004). Hence, the proximity of the experimenter in the no barrier conditions might have shortened the duration of eye contact. However, because of the notable inter-individual differences, data on a larger group of animals would be needed to critically scrutinize this possibility.

We then compared the aversion latencies between animals. In the barrier condition, Fin reacted significantly faster than all the other monkeys (Kruskal–Wallis test with Bonferroni correction, *p* < 0.0001), and Flo reacted significantly faster than Fer (*p* = 0.05) and Mir (*p* = 0.015). In the no barrier condition, Fin and Flo were still the monkeys with the fastest reactions (*p* < 0.0001), but did not significantly differ from each other (*p* = 1).

**Fig. 4:**
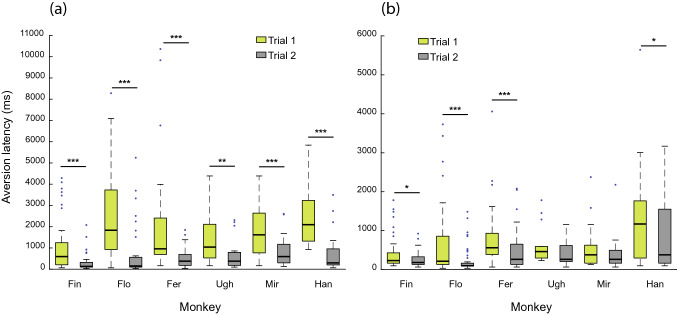
Comparison of head-gaze aversion latencies between trials 1 and trials 2, in the barrier (**a**) and non-barrier (**b**) condition. Eye contact duration in trial 1 was consistently longer than that in trial 2. Only data for Ugh and Mir showed no significant difference between type of trial in the no barrier condition. * p < 0.05, ** *p* < 0.01, *** p < 0.001

Prompted by the observation that the first period of eye contact of each session, namely at the beginning of each play cycle, was longer than that in the consecutive trial, we took a closer look at the dependency of gaze aversion latencies on trial number. When trial 1 was followed by trial 2, the duration of first eye contact was generally longer than the second one following the first head-gaze aversion, in both experimental conditions and for each individual animal, with only two exceptions in the no barrier condition (Wilcoxon signed-rank test; Fig. 4). For sequences of three or more trials, further shortening of the duration of eye contact was only shown by Flo, between trials 2 and 3.

### Head-cocking influences head-gaze aversion latencies

As shown in Fig. 1c, some animals, before turning their heads away, responded to eye contact with head-cocking (see Supplementary file 3 for examples), defined here as rotation of the observer´s head around a relatively fixed naso-occipital axis, in this case directed at the experimenter’s eyes. The rotation was exhibited either clockwise or counterclockwise, without a significant difference between the two. Previous reports of head-cocking in marmosets and other primates (Rogers et al. 1993; Kaplan and Rogers 2006; Cantalupo et al. 2002) described this movement as a fast saccadic counter rotation of the head back to the upright position. However, the head-cocking we recorded preceded gaze aversion and did not involve an intermediate return of the head to the upright position.

**Fig. 5:**
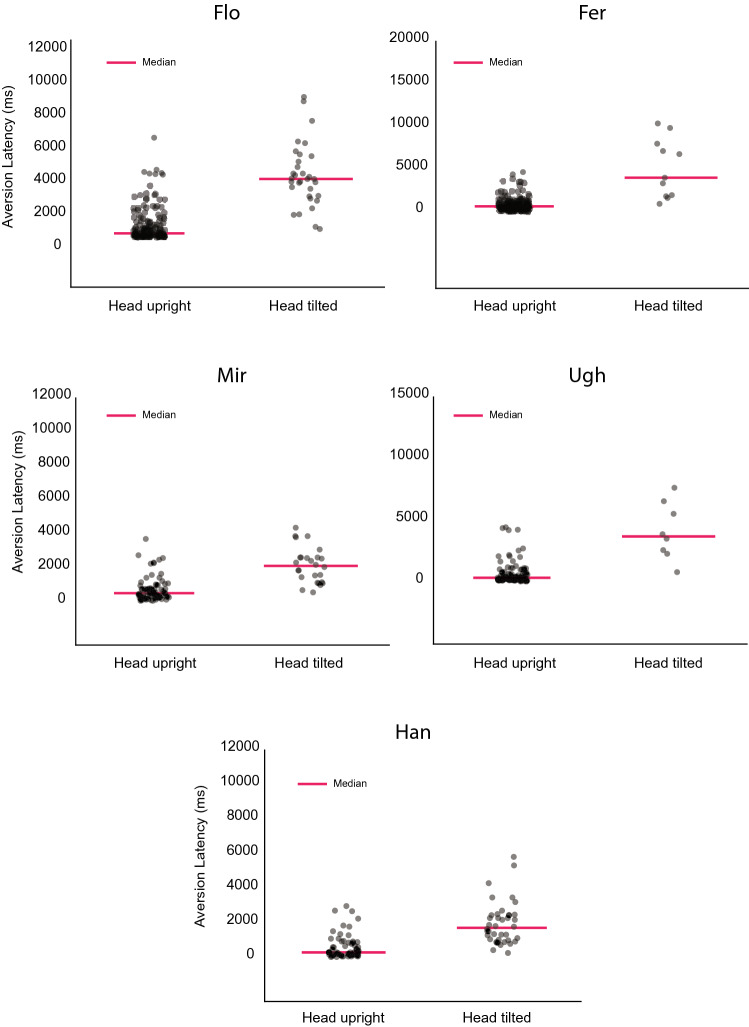
Maintaining eye contact with the head deviating from the upright position boosts eye contact duration. Comparison of latencies between simple aversion trials (head maintained in an upright position) and head-cocking trials (head tilted)

To understand whether tilting the head from the upright position influenced eye contact duration, we compared the aversion latencies of simple aversion trials, where the head was maintained in an upright position until it was turned with trials in which the animal performed head-cocking or established eye contact where the position of the head already deviated from the upright position. For group 1, this analysis was restricted to the barrier condition, in which the animals performed a larger number of head rotations. When the animals performed head-cocking or engaged in eye contact where the head was already tilted, eye contact was maintained for a significantly longer time compared to the simple aversion trials (Wilcoxon signed-rank test, Flo, *z* = − 4.937, *p* < 0.0001; Fer, *z* = − 2.312, *p* < 0.05; Ugh, *z* = -2.240, *p* < 0.05; Mir, *z* = − 4.280, *p* < 0.0001; Han, *z* = − 5.397, *p* < 0.0001; see Fig. 5 for group 1 and Supplementary Fig. 3 for group 2). Moreover, head-cocking followed eye contact with a short period of latency (see Supplementary Tables 1 and 2 for individual animal’s data) suggesting that eye contact was the critical event triggering this behavioral response rather than other visual factors.

## Discussion

The common marmosets consistently interrupted eye contact by means of stereotyped head-turning when interacting with the familiar experimenter who intermittently established eye contact with them (peekaboo game). Without doubt, looking at another individual’s face, whether they are a conspecific or human, and establishing eye contact is a rewarding urge for common marmosets, who have a strong liking for faces (Nummela et al 2019). Yet, our observations demonstrate that direct gaze can only be tolerated by common marmosets for a limited amount of time, even in a familiar affiliative interaction, and needs to be temporarily interrupted by looking away. 

Given that direct gaze is rarely a form of threat in this species, which is also supported by the fact that our animals exhibited overall behavioral signs of positive arousal (contact calls, remaining in the testing box, absence of aggressive behaviors towards the observer), it is very unlikely that gaze aversion was an attempt to evade a perceived threat or aggression, as seen in many other primate species. Rather, the urge to avert their gaze must have a different reason. As previously described, the dynamics of our experiment resemble those of peekaboo, a game often played by caretakers with infants. Humans infants’ reactions to the sudden appearance of the face during peekaboo develop with age; they usually consist of smiling or cooing when they are very young (around 3 months of age), and then additionally consist of hiding the face (for example by covering it with a blanket or averting their gaze or turning their head) as they get older. We showed that the reaction of common marmosets to the sudden appearance of a human face is very similar. As outlined in the Introduction with respect to human infants, marmosets may also primarily break eye contact to cope with the emotional arousal elicited by a direct gaze, as even if this is not perceived as threatening or aggressive, it can still be experienced as emotionally overwhelming. Breaking eye contact repeatedly may help to limit the level of arousal with the fundamental advantage for the animal of being able to prolong the overall duration of the interaction. For this reason, we believe that gaze aversion may also function as a prosocial behavior, in that it allows prolonged social interaction if there is motivation for it. Moreover, the fact that marmosets exhibit a behavior strikingly similar to the one seen in human infants that still lack executive control, suggests that the disengagement is quasi-reflexively driven by subcortical structures, as a fast, protective mechanism against over-excitation. If our hypothesis that head–gaze aversion and head-cocking in marmosets serves to reset emotional arousal is correct, then we would expect correlated changes in measurements of accompanying physiological indicators of arousal such as heart rate. Such measurements were beyond the scope of the present work. However, they have been performed on human infants, and in their case provided clear support for the hypothesized link between gaze aversion and arousal reduction (Field 1981).

We think that the type of emotional bond between an animal and a familiar experimenter may be the main determinant of prosocial gaze aversion in this type of situation. A familiar experimenter will be associated with positive experiences, such as provision of food and treats, as well as play. His/her direct gaze will always signals positive intent, which provides the monkey with a strong motivation to interact, but also increases the animal’s level of arousal. A mechanism that restricts the level of arousal will allow for a longer duration of interaction, and enable avoidance of the flight reaction: fleeing from the excessive arousal. Arguably, the behavior of the experimenter typical of peekaboo which was used in the present study to interact with the animals, in which the marmosets could see her face and direct gaze only for distinct periods of time interrupted by pauses, helped to prevent a flight reaction of the animals and to maintain their interest due to the reward of seeing the experimenter’s face and eyes. Even though we did not test the animals in interaction with an unfamiliar experimenter, previous studies have established that common marmosets present a typical flight reaction when experiencing an unfamiliar individual who stares at them for a relatively long period of time [intruder test (Quah et al. 2020; Alexander et al. 2020)]. In this latter condition, experienced as threatening and dangerous rather than rewarding, an unbridled increase in arousal is certainly advantageous as it will elicit an early flight reaction, which is potentially vital to the animal’s survival. This reaction is also accompanied by alarm calls, head and body bobs, and the avoidance of space closer to the other individual, spending more time at the back of the cage, which is a behavioral pattern that is overall very different from the one during gaze aversion in our experiment.

The alternation of eye contact with gaze aversion ended after a few rounds of peekaboo. This may indicate that arousal levels can only be somewhat limited by gaze aversion and may accumulate over time, finally making it necessary for the monkey to keep distance from the person interacting with them. Indeed, the idea of an incomplete arousal reset is supported by our finding that initial eye contact in a given session is always longer in duration than in subsequent trials. Although a temporary decline in interest in the other individual cannot be excluded, we suggest that the animal’s interest in the interaction must be rapidly restored, as we did not observe any long-term changes in the attractivity of the human agent for the marmoset.

One may wonder if the head-gaze aversion that characterizes the interaction between a marmoset and a familiar human has relevance for interactions between marmosets in the absence of human interference. Indeed, we observed head-gaze aversion as elements of interactions of monkeys with conspecifics in the following contexts:

1. During food competition, when a subordinate animal, looking at a treat held by a dominant monkey, averts its gaze as soon as the dominant animal engages in eye contact.
2. When a monkey is reunited with familiar animals following a separation of around 2 weeks’ duration.

The latter configuration is reminiscent of that between familiar humans, and this behavior may thus be interpreted as an example of natural gaze aversion. The interpretation of the former configuration is less straightforward. Here an attractor, who arguably induces positive emotions due to holding the treat, is present. On the other hand, the dominant monkey in possession of the treat will hardly be expected to be willing to share, and perhaps will even be perceived as threatening. Hence, gaze aversion in this case might be more similar to the standard agonistic pattern exhibited by other nonhuman primates. Moreover, the subordinate monkey, by averting his/her gaze, will signal disinterest in the treat, and thus avoid conflict.

### Head-cocking: a behavioral strategy to cope with eye contact?

The longest eye contact durations were registered when the animals looked at the experimenter, while assuming a head-cocking position before averting their gaze. Does this observation suggest that head-cocking may help to boost tolerance to eye contact? Head-cocking has previously been described as a stereotyped behavior exhibited by a large number of simians and prosimians (Menzel 1980), as well as by quite a few non-primate species (owls, dogs). Common marmosets are known to cock their head in reaction to the appearance of new objects (e.g., flies, pieces of food), or other individuals (“head-cock staring”) like cage mates or human strangers (Menzel and Menzel 1980). It is more frequent when directed towards living beings (Cantalupo et al. 2002) and it gradually decreases in frequency with age (Kaplan and Rogers 2006). The functional significance of head-cocking in primates remains unclear. Time and again it has been suggested to facilitate the scrutiny of objects, in particular novel ones, by improving visual capacity (Kaplan and Rogers 2006; Menzel 1980; Menzel and Menzel 1980). Yet, the visual mechanism that might underpin this presumed role of head-cocking in object analysis has remained elusive, and experimental evidence supporting a role in visual perception has, to the best of our knowledge, never been presented. In our analysis, it emerged that head-cocking significantly prolonged the duration of eye contact with the experimenter. Hence, could it be that head-cocking helps to decrease the emotional impact of the other’s face and eyes, thereby allowing longer periods of direct gaze? We and other primates may quickly detect horizontally oriented eyes because of experience-dependent fine-tuning of the visual system. By the observer rotating their head, the retinal image of the eyes will be tilted relative to the horizontal, arguably compromising perception of the gestalt and consequently reducing its emotional impact. The fact that both humans and lemurs exhibit longer fixation duration when exposed to tilted faces or tilted dots resembling eyes [humans (Coss 1979; Davidenko et al. 2019); lemurs (Coss 1978)] is clearly in line with the assumption that image tilt may reduce the impact of the other´s eyes. However, head-cocking may not only serve to regulate the animal’s level of arousal, but may also help to stabilize communication by heralding a more abrupt disengagement based on head-gaze aversion. In any case, we emphasize that unfamiliarity with the human agent responsible for triggering head-cocking, as suggested by previous work (De Boer et al. 2013), can be excluded in our experimental context, as the animals were used to seeing the same agent day after day and did not exhibit a decrease in head-cocking frequency over time.

Finally, it is worth mentioning that the analysis of behavioral reactions during social games is a useful tool for the detection of alterations in social behavior in early childhood (Koegel et al. 2014; Hakuno et al. 2018). In particular, children with autism spectrum disorders (ASD) show altered engagement in such social games. One hallmark of ASD, a complex neurodevelopmental disorder whose neural foundations remain largely unknown, is a compromised ability to engage in eye contact, with a tendency to evade the other’s gaze. We believe, therefore, that our findings, namely the fact that common marmosets engage in social games with a human experimenter and also exploit gaze aversion, strengthen the hope that this species could serve as a suitable model for the study of the foundations of social interactions and their alterations in ASD.

### Conclusions

We described a stereotypical head-gaze aversion behavior of common marmosets when engaging in direct eye contact with a human experimenter during a playful interaction. While gaze aversion is a well-established means of evading interaction with a subject considered threatening or aversive, the same behavior may be used to limit the impact of potentially overwhelming positive emotions elicited by a familiar face. Breaks from direct eye contact, arguably associated with an abatement of excessive arousal, allow the animal to maintain an interaction that is socially rewarding and holds the promise of further rewards. Head-cocking in view of the other individual may also limit the arousal associated with the social interaction, as tilting the head alters how the other individual’s direct gaze is perceived.

Human infants use head-gaze aversion as a regulator of perceptual input, both during stressful and positive interactions. We have shown here that common marmosets, whose evolutionary path started to deviate from that of humans around 35 million years ago (Miller et al. 2016), display a strikingly similar behavior in a familiar context. This suggests the possibility of a shared and conceivably homologous function of head-gaze aversion, opening up the prospect of using marmosets as a model system in future work on the neural architecture of a presumably conserved behavior.

## Supplementary Information

Below is the link to the electronic supplementary material.

Supplementary file1 (PDF 679 KB)

Supplementary file2 (AVI 1933 KB)

Supplementary file3 (AVI 998 KB)

Supplementary file4 (AVI 499 KB)

## Data Availability

The datasets generated during and/or analyzed during the current study are available from the corresponding authors on reasonable request.

## References

[CR1] Alexander L, Wood CM, Gaskin PLR, Sawiak SJ, Fryer TD, Hong YT, McIver L, Clarke HF, Roberts AC (2020). Over-activation of primate subgenual cingulate cortex enhances the cardiovascular, behavioral and neural responses to threat. Nat Commun.

[CR2] Argyle M, Dean J (1965). Eze-contact, distance and affiliation. Sociometry.

[CR3] Bard KA, Myowa-Yamakoshi M, Tomonaga M, Tanaka M, Costall A, Matsuzawa T (2005). Group differences in the mutual gaze of chimpanzees (*Pan troglodytes*). Dev Psychol.

[CR4] Burkart JM, Heschl A (2006). Geometrical gaze following in common marmosets (*Callithrix jacchus*). J Comp Psychol.

[CR5] Calhoun JB (1962). Population density and social pathology. Sci Am.

[CR6] Cantalupo C, McCain D, Ward JP (2002). Function of head-cocking in Garnett’s greater bush baby (*Otolemur garnettii*). Int J Primatol.

[CR7] Chance MRA (1962). An interpretation of some agonistic postures: the role of “cut-off” acts and postures. Symp Zool Soc Lond.

[CR8] Chen HC, Kaplan G, Rogers LJ (2009). Contact calls of common marmosets (*Callithrix jacchus*): influence of age of caller on antiphonal calling and other vocal responses. Am J Primatol.

[CR9] Cohn JF, Tronick EZ (1983). Three-month-old infants' reaction to simulated maternal depression. Child Dev.

[CR10] Cohn FJ, Tronick EZ (1987). Mother-infant face-to-face interaction: the sequence of dyadic states at 3, 6, and 9 months. Dev Psychol.

[CR11] Cook M (1977). Gaze and mutual gaze in social encounters. Am Sci.

[CR12] Coss RG (1978). Perceptual determinants of gaze aversion by the lesser mouse lemur (*Microcebus murinus*), the role of two facing eyes. Behaviour.

[CR13] Coss RG (1979). Perceptual determinants of gaze aversion by normal and psychotic children: the role of two facing eyes. Behaviour.

[CR14] Coss RG, Marks S, Ramakrishnan U (2002). Early environment shapes the development of gaze aversion by wild bonnet macaques (*Macaca radiata*). Primates.

[CR15] Davidenko N, Kopalle H, Bridgeman B (2019). The upper eye bias: rotated faces draw fixations to the upper eye. Perception.

[CR16] De Waal FBM, Yoshihara D (1983). Reconciliation and re-directed affection in rhesus monkeys. Behaviour.

[CR17] De Boer RA, Overduin-de Vries AM, Louwerse AL, Sterck EH (2013). The behavioral context of visual displays in common marmosets (*Callithrix jacchus*). Am J Primatol.

[CR18] Ehardt CL, Blount BG (1984). Mother–infant visual interaction in Japanese macaques. Dev Psychobiol.

[CR19] Evinger C, Manning KA, Pellegrini JJ, Basso MA, Powers AS, Sibony PA (1994). Not looking while leaping: the linkage of blinking and saccadic gaze shifts. Exp Brain Res.

[CR20] Ferrari PF, Paukner A, Ionica C, Suomi SJ (2009). Reciprocal face-to-face communication between rhesus macaque mothers and their newborn infants. Curr Biol.

[CR21] Field T (1981). Infant gaze aversion and heart rate during face-to-face interactions. Infant Behav Dev.

[CR22] Field T, Vega-Lahr N, Scafidi F, Goldstein S (1986). Effects of maternal unavailability on mother–infant interactions. Infant Behav Dev.

[CR23] Hakuno Y, Pirazzoli L, Blasi A, Johnson MH, Lloyd-Fox S (2018). Optical imaging during toddlerhood: brain responses during naturalistic social interactions. Neurophotonics.

[CR24] Helminen TM, Kaasinen SM, Hietanen JK (2011). Eye contact and arousal: the effects of stimulus duration. Biol Psychol.

[CR25] Hietanen JK (2018). Affective eye contact: an integrative review. Front Psychol.

[CR26] Hinde RA, Rowell TE (1962). Communication by postures and facial expression in the rhesus monkey (*Macaca mulatta*). Proc Zool Soc Lond.

[CR27] Kano K, Hirata S, Call J (2015). Social attention in the two species of* Pan*: bonobos make more eye contact than chimpanzees. PLoS One.

[CR28] Kaplan G, Rogers LJ (2002). Patterns of gazing in orangutans (*Pongo pygmaeus*). Int J Primatol.

[CR29] Kaplan G, Rogers LJ (2006). Head-cocking as a form of exploration in the common marmoset and its development. Dev Psychobiol.

[CR30] Kleinke CL (1986). Gaze and eye contact: a research review. Psychol Bull.

[CR31] Koegel L, Singh A, Koegel R, Hollingsworth J, Bradshaw J (2014). Assessing and improving early social engagement in infants. J Posit Behav Interv.

[CR32] Maestripieri D (1997). Gestural communication in macaques: usage and meaning of nonvocal signals. Evol Commun.

[CR33] Mendelson MJ (1982). Visual and social responses in infant rhesus monkeys. Am J Primatol.

[CR34] Mendelson MJ, Haith MM, Goldman-Rakic PS (1982). Face scanning and responsiveness to social cues in infant rhesus monkeys. Dev Psychol.

[CR35] Menzel CR (1980). Head-cocking and visual perception in primates. Anim Behav.

[CR36] Menzel CR, Menzel EW (1980). Head-cocking and visual exploration in marmosets,* Saguinus fuscicollis*. Behaviour.

[CR37] Miller CT, Freiwald WA, Leopold DA, Mitchell JF, Silva AC, Wang X (2016). Marmosets: a neuroscientific model of human social behavior. Neuron.

[CR38] Miss FM, Burkart JM (2018). Corepresentation during joint action in marmoset monkeys (*Callithrix jacchus*). Psychol Sci.

[CR39] Mitchell JF, Leopold DA (2015). The marmoset monkey as a model for visual neuroscience. Neurosci Res.

[CR40] Nichols KA, Champness BG (1971). Eye gaze and the GSR. J Exp Soc Psychol.

[CR41] Nummela SU, Jutras MJ, Wixted JT, Buffalo EA, Miller CT (2019). Recognition memory in marmoset and macaque monkeys: a comparison of active vision. J Cogn Neurosci.

[CR42] Pandey S, Simhadri S, Zhou Y (2020). Rapid head movements in common marmoset monkeys. iScience.

[CR43] Plutchik R (1980). Emotion: a psychoevolutionary synthesis.

[CR44] Quah SKL, Cockcroft GJ, McIver L, Santangelo AM, Roberts AC (2020). Avoidant coping style to high imminence threat is linked to higher anxiety-like behavior. Front Behav Neurosci.

[CR45] Reddy V (2000). Coyness in early infancy. Dev Sci.

[CR46] Redican WK (1975). Facial expressions in nonhuman primates.

[CR47] Robson KS (1967). The role of eye-to-eye contact in maternal-infant attachment. J Child Psychol Psychiatry.

[CR48] Rogers LJ, Stafford D, Ward JP (1993). Head cocking in galagos. Anim Behav.

[CR49] Sato N, Nakamura K (2001). Detection of directed gaze in rhesus monkeys (*Macaca mulatta*). J Comp Psychol.

[CR50] Stifter CA, Moyer D (1991). The regulation of positive affect: gaze aversion activity during mother-infant interaction. Infant Behav Dev.

[CR51] Tada H, Omori Y, Hirokawa K, Ohira H, Tomonaga M (2013). Eye-blink behaviors in 71 species of primates. PLoS One.

[CR52] Tronick EZ (1989). Emotions and emotional communication in infants. Rev Am Psychol.

[CR53] Wang A, Payne C, Moss S, Jones WR, Bachevalier J (2020). Early developmental changes in visual social engagement in infant rhesus monkeys. Dev Cogn Neurosci.

[CR54] Yamagiwa J (1992). Functional analysis of social staring behavior in an all-male group of mountain gorillas. Primates.

[CR55] Yorzinski JL (2016). Eye blinking in an avian species is associated with gaze shifts. Sci Rep.

