# Supplementary material for: A prosocial function of head-gaze aversion and head-cocking in common marmosets
Source: Primates. 2022 Jul 15;63(5):535–46. doi: 10.1007/s10329-022-00997-z (PMC9463209; doi:10.1007/s10329-022-00997-z)
Supplement: Supplementary file 1 — Supplementary file1 (PDF 679 KB) [file 10329_2022_997_MOESM1_ESM.pdf]

SUPPLEMENTARY INFORMATION

A PROSOCIAL CHARACTER OF HEAD-GAZE AVERSION AND HEAD-COCKING IN COMMON MARMOSETS

S. Spadacenta[1]*, P.W. Dicke[1], P. Thier[1]*

[1]Cognitive Neurology laboratory, Hertie Institute for Clinical Brain Research, University of Tübingen

*for correspondence: silvia.spadacenta@uni-tuebingen.de, thier@uni-tuebingen.de

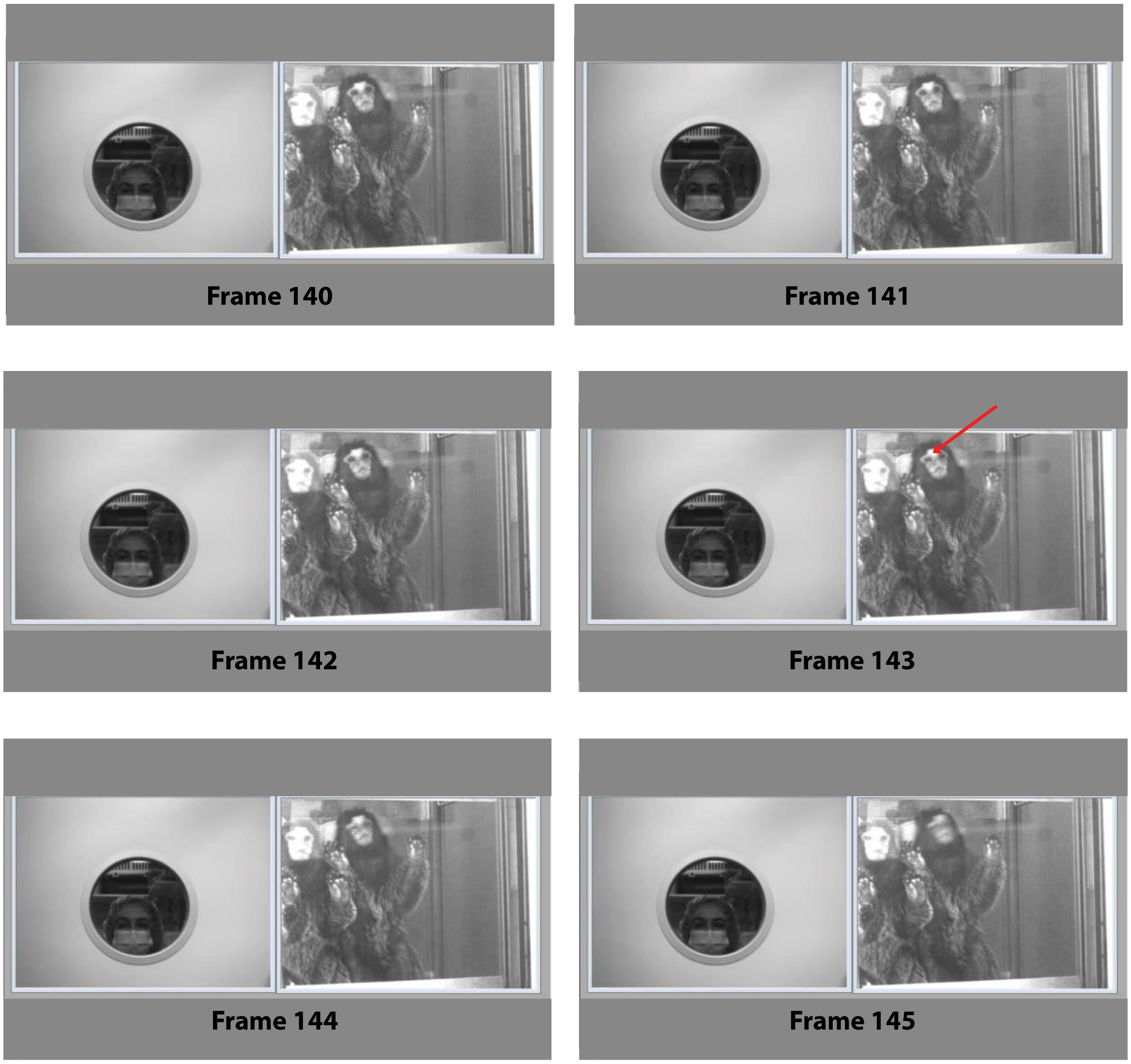

Supplementary Fig. 1 Sample of frames extracted from one video example (Barrier condition). In this case the head/gaze aversion start event for the animal on the right side (experimenter view) is identified from frame 143, where the marmoset's left eye starts to close (red arrow). The

subsequent frame (144) shows a clear full closure of the eyes and the head moving towards the animal's left side. Note that the animal on the right side reacted later in subsequent frames.

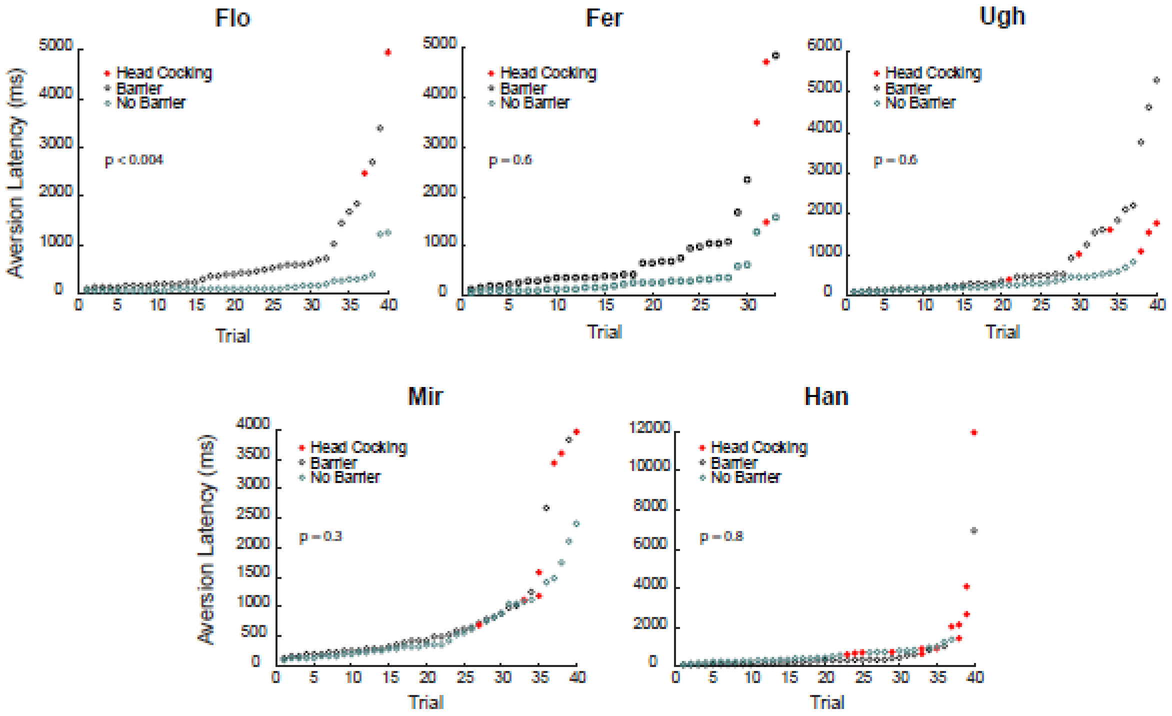

Supplementary Fig. 2 Effect of experimental condition on aversion latency for 5 animals (Flo, Fer, Ugh, Mir, Han) interacting with a second familiar experimenter. For each monkey we show the single trial aversion latency sorted by ascending duration. Red dots highlight trials in which the animals either performed a head-cocking or were looking at the experimenter with a tilted head position from the start of eye contact. The resulting statistics comparing barrier and non-barrier condition latencies with Mann-Whitney-U-test are reported.

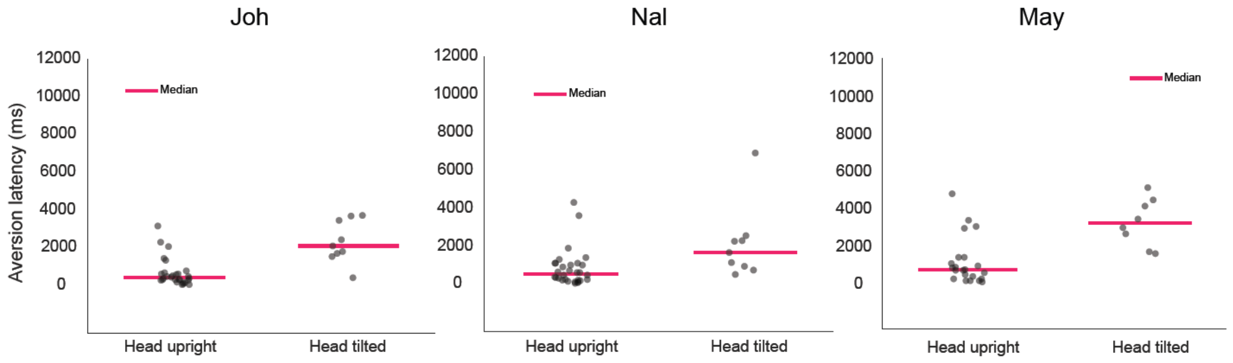

Supplementary Fig. 3 Comparison between aversion latencies of simple aversion trials and head-cocking trials (Monkey Joh, Nal, May of group 2). As for group 1, eye contact was maintained for a longer duration when the animals head position was tilted (Wilcoxon signed-rank test, Joh, zval = -2.521, p = 0.01; Nal: zval = -2.073, p <0.03; May: zval = -1.96, p < 0.05). Each dot corresponds to a single trial latency.

Monkey	Fin		Flo		Fer		Ugh		Mir		Han	
**Condition**	Barrier	No Barrier	Barrier	No Barrier	Barrier	No Barrier	Barrier	No Barrier	Barrier	No Barrier	Barrier	No Barrier
Median aversion latency (ms)	198	198	462	132	627	495	428.5	429	759	396	891	693
Blinks (%)	81.5	69	69.5	86.5	75	60.5	35	25.5	14	13.5	33	24.5
Vocalizations (%)	22.5	8	5	12.5	9	11.5	4	7	12	9	4	2
Body moved (%)	0	0	15	25	9	20.5	1	1	1	4	14	12
Head- cocking (%)	0	0	16	1.5	5.5	2	3	3	25	2	24	8
Median head-cocking start latency (ms)	-	-	198	1947	990	676.5	957	462	594	1666	429	907.5

Supplementary table 1. Behavioral values summary of group 1 monkeys.

Monkey	Ali	Joh	Nal	Sis	May	Dai	Que	Eva	Wil	Giz
**Condition**	No Barrier	No Barrier	No Barrier	No Barrier	No Barrier	No Barrier	No Barrier	No Barrier	No Barrier	No Barrier
Median aversion latency (ms)	198	528	726	297	957	1023	462	132	577.5	198
Blinks (%)	84	78,4	69	54.3	67	27.01	37.1	90	54.2	69.6
Vocalizations (%)	13	0	7.7	8.6	20	2.8	20	0	4.2	0
Body moved (%)	13	16.2	3	0	0	0	0	10	17	26.1
Head- cocking (%)	0	24.3	23.1	8.6	27	16.2	2.8	0	16.6	4.34
Median head-cocking start latency (ms)	-	528	429	561	511	297	495	-	165	495

Supplementary table 2. Behavioral values summary of group 2 monkeys.

Monkey	Flo		Fer		Ugh		Mir		Han	
**Condition**	Barrier	No Barrier	Barrier	No Barrier	Barrier	No Barrier	Barrier	No Barrier	Barrier	No Barrier
Median aversion latency (ms)	412.5	99	429	231	379.5	264	462	363	330	495
Blinks (%)	75	82.5	82	61	70	72.5	47.5	27.5	72.5	50
Vocalizations (%)	2.5	0	6.1	9.1	2.5	2.5	10	2.5	2.5	0
Body moved (%)	7.5	52.5	6.1	24.4	0	2.5	15	5	2.5	5
Head- cocking (%)	5	0	3	0	0	5	10	0	10	5
Median head-cocking start latency (ms)	891	-	-	-	-	181.5	1551	-	462	379.5

Supplementary table 3. Behavioral values summary of group 1 monkeys tested by a second experimenter.
